# Hepatitis C Virus infections trends in Italy, 1996-2006

**DOI:** 10.5812/kowsar.1735143X.767

**Published:** 2011-11-30

**Authors:** Giuseppe La Torre, Maria Rosaria Gualano, Leda Semyonov, Nicola Nicolotti, Walter Ricciardi, Antonio Boccia

**Affiliations:** 1 Department of Public Health and Infectious Diseases, Sapienza University of Rome, Rome, Italy; 2 Institutes of Hygiene, Catholic University of the Sacred Heart, Rome, Italy

**Keywords:** Hepatitis C, Infection, Trend, Italy

## Abstract

**Background:**

The World Health Organization (WHO) estimates that about 180 million people, 3% of the world population, are infected with the hepatitis C virus (HCV). In Italy, the prevalence in the general population is reported to be greater than 5% and 9% among households of HCV-positive patients.

**Objectives:**

The aim of this study was to estimate the trends of HCV infection in Italy in the period 1996–2006.

**Materials and Methods:**

The formula ln (rate) = b × years was applied for logarithmic transformation of the incidence rates to obtain time trends of HCV infection, using the join-point regression program software version 3.3.1. Linear graphs representing trends and the annual percentage change (APC) were considered for each joinpoint. Time changes are expressed as expected annual percentage change (EAPC) with the respective 95% confidence intervals (CIs); significance levels of time trends are also reported. The null hypothesis was tested using a maximum of 3 changes in slope with an overall significance level of 0.05 divided by the number of joinpoints in the final model.

**Results:**

Considering all age groups, the incidence rate decreased from 2.02 to 0.55 per 100,000. The join-point analysis showed a statistically significant decrease in the incidence rates of HCV infection. No join-points were found in any age groups. Our data show that the incidence rates of HCV infections have considerably decreased in each age group throughout the studied period (1996–2006).

**Conclusions:**

This decreasing trend in HCV infections is, in part, attributable to behavioral and social changes. Improved hygiene, use of precautions in medical settings, blood screening, and sexual educational campaigns seem to have contributed to reduce the transmission of infection during the last 10 years.

## 1. Background

Hepatitis C virus (HCV) infection is a major public health problem and chronic liver disease secondary to HCV is a leading indication for liver transplantation [1]. In fact, infection with HCV is generally asymptomatic and in less than half of the infection cases, hepatitis becomes chronic, progressing to cirrhosis or liver cancer [2][3]. The disease burden of hepatitis C is high. The World Health Organization (WHO) estimates that about 180 million people, 3% of the world population, are infected with HCV; of these, 130 million are chronic HCV carriers. Three to four million people are newly infected each year [2]. Disease prevalence is low (< 1%) in Australia, Canada, and Northern Europe; it is approximately 1% in countries of medium endemicity, such as the USA and most of Europe, and high (> 2%) in many countries in Africa, Latin America, and Central and Southeastern Asia. In these countries, the prevalence of HCV infection could increase to 10% [2]. Currently, the majority of HCV infections occur among adults, and injection drug use was the most common risk factor [4].

At present, this infection is not eradicable, as a vaccination against hepatitis C is not yet available. Acquisition of HCV infection typically involves the parenteral route (transfusions of blood or blood products from unscreened donors, injection drug use, or unsafe therapeutic injections), but HCV can also be transmitted by occupational injury (contaminated needles or sharp instruments), hemodialysis, and tattooing [5]. Moreover, dental care was found to be associated with HCV seropositivity [6]. In addition, at least 50% of all HCV-positive patients do not have a history of blood transfusion or exposure to any other parenteral risk factor [7]. In fact, familiar clustering of HCV infection has been demonstrated in epidemiological studies, but it is still controversial whether HCV can be transmitted to partners or other household contacts through horizontal transmission [8][9]. Since the late 1980s, the incidence of acute hepatitis C has also declined. In particular, with regard to HCV cases associated with transfusion, this decrease could be attributed to the requirement for blood screening worldwide [4].

However, it is estimated that mortality related to HCV infection (death from liver failure or hepatocellular carcinoma) will continue to increase over the next 2 decades [9]. In Italy, the incidence of HCV has decreased from 5 per 100,000 in 1985 to 1 per 100,000 in 1996 [10]. Mariano et al. [11], using mathematical modeling in order to estimate the HCV burden from 1950 to 2030, predicted that the number of individuals with HCV-related cirrhosis and HCV liver-related deaths will be halved by 2025.

## 2. Objectives

The aim of this study was to estimate the trends of HCV infection in Italy in the period 1996-2006 for the whole population, as well as for gender and different age groups, using the joinpoint regression method

## 3. Materials and Methods

Incidence rates of HCV infection (per 100,000) were calculated using data of cases of the integrated epidemiological system for acute viral hepatitis in Italy (SEIEVA) of the Italian Ministry of Health [12] and population data from the Health for All Database of the Italian National Institute of Statistics (ISTAT) [13], for the period 1996-2006. Data regarding incidence rates were stratified by gender and age groups (in years: 0-14; 15-24; 25-64; ≥ 65). In Italy, a ministerial decree of December 15, 1990, states the mandatory registration of illnesses that could constitute an epidemiological risk. Since 1993, data on frequency of some infectious diseases (including HCV infections) were published by the Italian Ministry of Health in the Italian Epidemiology Bulletin [13]. Data are shown by year and specific age group. ISTAT is a public and independent research organization that operates in continuous interaction with the academic and scientific communities. Since 1926, it is the main producer of official statistics in Italy, available for citizens, researchers, and policy-makers. Data on the resident population are extrapolated from the registry office records of Italian municipalities.

### 3.1. Statistical Analysis

Rates were computed as number of cases per general population, as previously described. In order to obtain time trends of HCV infection, the following formula [14] was applied for logarithmic transformation of incidence rates:

ln (y) = b × x,

Where "x" represents the calendar years, "b" is the regression coefficient, and "y" the incidence rate. In particular, a joinpoint represents the time point when a significant trend change is detected. Time changes are expressed as expected annual percentage change (EAPC) with the respective 95% confidence interval (95% CI); significance levels of time trends are also reported. The null hypothesis was tested using a maximum of 3 changes in slope with an overall significance level of 0.05 divided by the number of joinpoints in the final model. Linear graphs were created to represent trends. Statistical analysis was conducted by using the joinpoint regression program software version 3.3.1. The Poisson model was applied to control heteroskedasticity in the population.

## 4. Results

**Fig 1:**
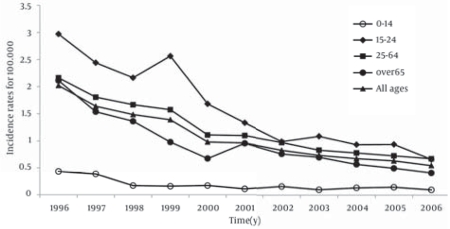
Incidence Rates of Hepatitis C Virus (HCV) Infections per100,000 in Italy, 1996-2006 (Linear Scale)

A total number of 6,806 cases of HCV infection occurred during the period 1996-2006, and a strong reduction in the incidence rates of HCV infection was observed in all age groups (Figure 1). In particular, in 1996, the highest incidence rate of HCV infection was found in subjects aged from 15 to 24 years (2.97 per 100,000), followed by the 25-64 years age group (2.16 per 100,000). In the period 1996-2006, the incidence rate decreased from 0.43 to 0.10 per 100,000 in the age group 0-14 years, from 2.97 to 0.66 in the group 15-24 years, from 2.16 to 0.67 in the age group 25-64 years, and from 2.11 to 0.41 in subjects aged 65 years or older. Considering all age groups, the incidence rate decreased from 2.02 to 0.55 per 100,000.

Trend analysis showed a statistically significant decrease in the incidence rates of HCV infection in all age groups. The most remarkable decrease was noted among the male population. In this group, in the period 1996-2006, the incidence rate decreased from 0.59 to 0.05 per 100,000 in the age group 0-14 years, from 4.25 to 0.90 in the group 15-24 years, from 2.81 to 0.88 in the age group 25-64 years, and from 2.64 to 0.45 in subjects aged 65 years or older. However, in the female population for the same time period, the incidence rate changed from 0.27 to 0.15 per 100,000 in the age group 0-14 years, from 1.64 to 0.41 in the group 15-24 years, from 1.53 to 0.47 in the age group 25-64 years, and from 1.75 to 0.38 in subjects aged 65 years or older. The EAPCs were -13.57 (95% CI: -18.92; -7.88) for the 0-14 age group; -13.57 (95% CI: -16.37; -10.67) for the 15-24 age group; -11.6 (95% CI: -12.93; -10.26) for the 25-64 age group; and -14.29 (95% CI: -17.14; -11.34) for subjects aged 65 years or older (Table 1). The overall annual decrease was -12.45% (P < 0.001). No joinpoints were found in any age groups. Trend analyses by age group are shown inFigure 2 Figure 3 Figure 4 Figure 5 Figure 6

**Table 1:** Results of the Joinpoint Regression Analysis by Age Group and Gender during the Period 1996-2006

	**EACP^a^ %**	**95% CI^a^**	**P value**
**Male**			
0–14	-16,58	(-23.78; -8.69)	< 0.01
15–24	-14,2	(-16.91; -11.31)	< 0.001
25–64	-11,07	(-12.65; -9.46)	< 0.001
≥ 65	-13,95	(-17.95; -9.75)	< 0.001
All age groups	-12,23	(-13.51; -10.93)	< 0.001
**Female**			
0–14	-7,78	(-14.29; -0.77)	0.03
15–24	-11,68	(-16.42; -6.66)	< 0.001
25–64	-12,62	(-14.71; -10.47)	< 0.001
≥ 65	-14,67	(-18.12; -11.07)	< 0.001
All age groups	-12,8	(-14.86; -10.69)	< 0.001
**Total**			
0–14	-13,57	(-18.92; -7.88)	< 0.001
15–24	-13,57	(-16.37; -10.67)	< 0.001
25–64	-11,6	(-12.93; -10.26)	< 0.001
≥ 65	-14,29	(-17.14; -11.34)	< 0.001
All age groups	-12,45	(-13.76; -11.12)	< 0.001

^a^ Abbreviations: CI, confidence interval; EACP, estimated annual percentage change

**Fig 2:**
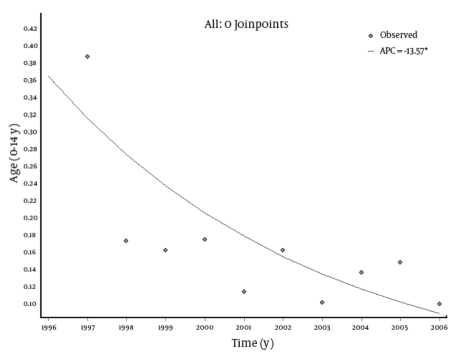
Trend of Incidence Rates (per 100,000) of Hepatitis C Virus HCV) Infections in Italy (Age: 0-14 Years), 1996-2006.APC = annual percentage change

**Fig 3:**
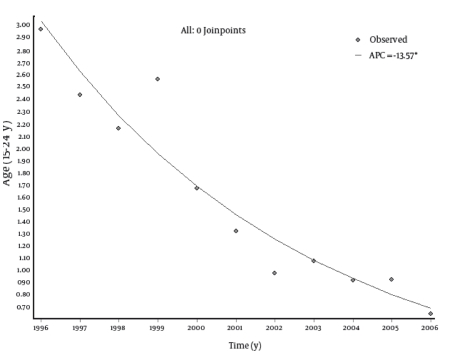
Trend of Incidence Rates (per 100,000) of Hepatitis C Virus (HCV) Infections in Italy (Age: 15-24 Years), 1996-2006 .APC = annual percentage change

**Fig 4:**
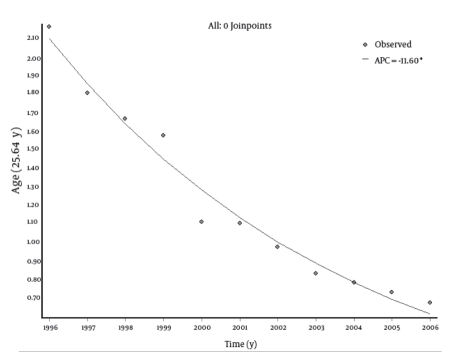
Trend of Incidence Rates (per 100,000) of Hepatitis C Virus (HCV) Infections in Italy (Age: 15-24 Years), 1996-2006 .APC = annual percentage change

**Fig 5:**
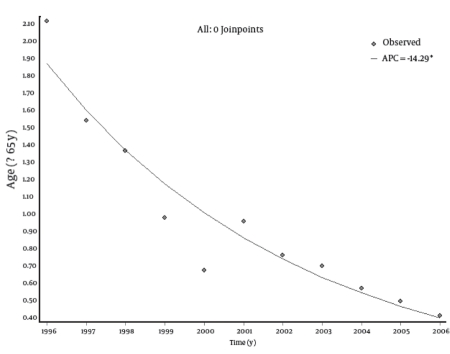
Trend of Incidence Rates (per 100,000) of Hepatitis C Virus (HCV) Infections in Italy (Age: 15-24 Years), 1996-2006 .APC = annual percentage change

**Fig 6:**
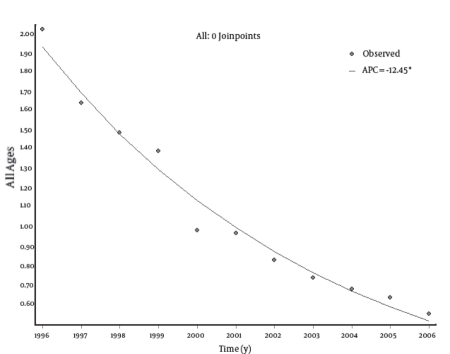
Trend of Incidence Rates (per 100,000) of Hepatitis C Virus (HCV) Infections in Italy (Age: 15-24 Years), 1996-2006 .APC = annual percentage change

## 5. Discussion

This study describes for the first time the trend of HCV infections in Italy after 1996 using the joinpoint regression method. Our study shows that in Italy, incidence rates of HCV infection have considerably decreased in each age group throughout the studied period (1996-2006). This trend is statistically significant, particularly among young people. In most European countries, the prevalence of HCV infection ranges from 0.5% to 2% [15]; whereas in Italy, a prevalence greater than 5% has been reported in some communities [16]. In addition, the prevalence of HCV infection is 9% among households of HCV-positive patients [17]. The strength of our study is that we examined HCV infection trends among the general Italian population for a 10-year period. In addition, while the majority of studies have described the epidemiology of HCV among specific groups of people, i.e. blood donors, drug users and health care workers [18][19][20][21][22][23][24][25][26][27][28][29][30][31][32][33][34], only a few studies have described the prevalence of HCV infections considering the general population [35][36][37][38][39][40][41][42]. The significant decrease in HCV infections underlines the behavioral changes that have taken place in recent years, and the special attention paid to sanitation during medical procedures, even for those performed at home. In fact, the current decrease in the incidence of HCV infection may be attributable to several factors, i.e. improved socio-economic conditions, smaller family size, introduction of compulsory screening for anti-HCV in blood banks, more sensitive procedures for HCV screening of blood donations and widespread use of disposable syringes [43].

Some limitations of this study have to be acknowledged. The estimates derived from the Health Information System could be affected by the open access to the health services and by the poor quality and irregularity of reporting. Therefore, data sources were critically assessed to take into account the possibility of under- or overestimation and to ensure that the reports were consistent with the epidemiological knowledge about diseases. At present, the residual risk of HCV transmission by transfusion is very low in industrialized countries; newly diagnosed cases of HCV infection could be limited to marginal risk factors, i.e. nosocomial infections, vertical transmission and use of intravenous drugs and beauty treatments. This supports the hypothesis that the prevalence of HCV infection will decrease significantly over the next 2 decades [44]. Identification of HCV-positive persons for appropriate counseling and management is the major focus of a national prevention program, and routine testing is recommended for persons likely to have an HCV infection [45]. Since an active immunization against HCV is still not available, precaution in medical settings, blood screening, sexual educational campaigns and hygiene campaigns for drug-addicts are the most powerful control measures necessary to reduce the prevalence of this infection, which represents a main goal of public health services.
